# Potentially Excessive Follow-up Blood Cultures in *Staphylococcus aureus* Bacteremia: Opportunities for Diagnostic Stewardship

**DOI:** 10.1093/ofid/ofag685

**Published:** 2026-10-29

**Authors:** Iris D N Van Bommel, Nienke L Plantinga, Maaike S M Van Mourik, Hanneke Boon, Stefan M T Vestjens, Marjolein P M Hensgens

**Affiliations:** Julius Centre for Health Sciences and Primary Care, University Medical Centre Utrecht, Utrecht University, Utrecht, The Netherlands; Department of Medical Microbiology and Immunology, St. Antonius Hospital, Nieuwegein, The Netherlands; Department of Medical Microbiology and Infection Control, University Medical Centre Utrecht, Utrecht, The Netherlands; Department of Medical Microbiology and Immunology, St. Antonius Hospital, Nieuwegein, The Netherlands; Department of Medical Microbiology and Immunology; Diakonessenhuis Hospital, Utrecht, The Netherlands; Julius Centre for Health Sciences and Primary Care, University Medical Centre Utrecht, Utrecht University, Utrecht, The Netherlands; Department of Infectious Diseases; University Medical Centre Utrecht, Utrecht, The Netherlands

**Keywords:** bacterial infections, blood cultures, diagnostic stewardship, follow-up, *staphylococcus aureus* bacteremia

## Abstract

**Background:**

Follow-up blood cultures are recommended in *Staphylococcus aureus* bacteremia (SAB) to document clearance, but excessive culturing increases patient burden and healthcare costs. Guidelines advise repeat cultures every 2–4 days after initiation of antibiotic treatment. We quantified follow-up blood culture practices and evaluated opportunities for diagnostic stewardship.

**Methods:**

We conducted a retrospective cohort study of adult SAB patients between 1 January 2014 and 1 January 2024 in 3 Dutch hospitals. A SAB episode comprised 30 days after the index culture and included a diagnostic period (≤2 days) and follow-up period (>2 days). Diagnostic stewardship opportunities during follow-up were defined as daily culturing, obtaining >2 sets per day, or continued culturing after clearance.

**Results:**

We identified 2499 SAB episodes among 2400 patients (median age 71 years, 36.8% female). Overall, 21014 blood culture sets were obtained, of which 12701 (60.4%) during follow-up. Follow-up cultures were performed in 83.4% of episodes. Among 6044 follow-up intervals, 3869 (64.0%) reflected daily culturing. Of 8037 follow-up culturing days, 302 (3.8%) had >2 sets per day. Among 1993 episodes with ≥1 negative culture, culturing continued beyond clearance in 903 (45.3%). Overall, 6432 of 12701 (50.6%) follow-up sets met ≥1 stewardship criterion; this proportion was higher in patients with endocarditis than in those without endocarditis in Hospital A.

**Conclusions:**

Follow-up blood cultures in SAB frequently exceeded guideline recommendations. Limiting follow-up to 2 blood culture sets every 2 days until clearance is an important opportunity for diagnostic stewardship, while more intensive culturing may be clinically indicated in selected patients.

## Teaser

This multicenter study focusing on Staphylococcus aureus bacteremia highlights opportunities to reduce excessive blood culturing during patient follow-up. Half of all follow-up blood culture sets met at least one potential diagnostic stewardship criterion, suggesting opportunities to optimize culturing frequency and duration while ensuring adequate documentation of bacterial clearance.

*Staphylococcus aureus* bacteremia (SAB) is a bloodstream infection with high morbidity and mortality [1, 2]. Blood culture sensitivity largely depends on the collected blood volume; therefore, 2 sets are recommended (1 aerobic and 1 anaerobic bottle each) to diagnose SAB [3–5]. For follow-up of SAB, the Infectious Diseases Society of America (IDSA) guideline on methicillin-resistant *S. aureus* (MRSA) [6] advises repeat blood cultures every 2–4 days after initiation of antibiotic treatment until bacterial clearance is documented [7, 8], and adherence with this metric is considered a key quality indicator [9, 10]. Dutch guidelines recommend obtaining 2 sets of follow-up blood cultures at least every 48 hours [11]. This practice serves to confirm eradication of the pathogen or to detect persistent bacteremia with subsequent diagnostic, prognostic, and therapeutic implications.

No standardized criteria exist to define a definitively negative culture, although a 5-day incubation period is internationally accepted [12–14]. Most *S. aureus* blood cultures turn positive early during incubation. Previous studies report that 95.5% to 97.3% of *S. aureus* cultures were detected within 48 hours [12, 15, 16].

Although repeat cultures are warranted, excessive blood culturing increases patient discomfort through phlebotomy and iatrogenic blood loss, adds unnecessary costs, contributes to the environmental impact associated with laboratory testing, and raises the risk of obtaining contaminated blood cultures. These factors have been associated with prolonged hospital stay and unnecessary antibiotic use [17–20]. Diagnostic stewardship therefore advocates targeted blood culture indications to optimize yield and minimize harm [21, 22].

This study was designed to investigate the hypothesis that follow-up blood cultures in patients with SAB are obtained more frequently and for longer than necessary, thereby creating opportunities for diagnostic stewardship. In this large multicenter study, we therefore quantified the timing, frequency, and duration of culturing for each episode of SAB.

## METHODS

### Study Design

We conducted a retrospective cohort study of SAB patients between 1 January 2014 and 1 January 2024 in 1 tertiary care hospital and 2 secondary care hospitals in the Netherlands.

### Study Population

Eligible patients were hospitalized adults (≥ 18 years) with ≥1 *S. aureus*-positive blood culture. Patients were excluded if they had objected to research use of their data. Patients with polymicrobial cultures were not excluded.

### Patient Consent Statement

The local institutional review boards of all participating hospitals approved the study. In accordance with Dutch legislation, the Medical Research Involving Human Subjects Act did not apply; therefore, formal ethical approval was waived (UMC Utrecht 24U-1444; St. Antonius Hospital Z25.041; Diakonessenhuis Hospital 2025–003).

### Data Collection

Retrospective data included patient age, sex, blood culture collection date, and microbiological results. From time zero (the index culture; see Table 1), all blood cultures within the 30-day episode window were included. Because collection times were reliable to the calendar day but not to clock times, analyses were based on calendar days. Time stamps were used solely to group bottles into sets. Source of infection, based on the diagnosis documented in the medical record by the treating physician, infectious diseases consultant, or clinical microbiologist, time to detection, and transfer data were available only for Hospital A, and mortality and hospital ward data for Hospitals A and C.

**Table 1.** Definition Overview

Item	Definition
Culturing day	A calendar day on which 1 or more blood culture bottles were collected. For analyses, the combined result of all bottles obtained on that day were used to define the day as positive (any bottle with microbial growth) or negative (all bottles without growth).
Set	A set of 2 simultaneously ordered blood culture bottles (1 aerobic and 1 anaerobic bottle if available; when only a single bottle was obtained at that time point, the single bottle was considered a set).
Positive *S. aureus* blood culture	A blood culture bottle with *S. aureus* growth.
Negative *S. aureus* blood culture	A blood culture bottle without *S. aureus* growth. Blood cultures yielding only non-*S. aureus* microorganisms were considered negative for *S. aureus*.
Microbiological concordance	Identical microbiological outcomes between a blood culture set and the immediately preceding set (ie, both negative or both positive for the same microorganism).
Index blood culture	The first positive blood culture bottle for *S. aureus.*
Episode	The 30-day period following the index blood culture.
Diagnostic period	≤2 days from the index blood culture (including the index blood culture), based on previously reported time to positivity [12, 15], reflecting initial diagnostic uncertainty.
Follow-up period	>2 days after the index blood culture.
Clearance	Two consecutive negative *S. aureus* culturing days.
Recurrence	A new positive blood culture bottle for *S. aureus* ≥2 days after at least 2 consecutive negative blood culturing days, and within 30 days of the index blood culture.
Skip phenomenon	An intermittent negative (N) culturing day between surrounding *S. aureus* positive (P) culturing days (P-N-P pattern), assessed within an episode.
Time to detection	The time between the blood culture incubation and the automated detection of bacterial growth, as recorded in the laboratory information system.

### Definitions

Table 1 summarizes all definitions. In this study, we focused specifically on diagnostic stewardship opportunities related to excess follow-up blood culturing, which were defined as follows:

1. **Repeating cultures daily**, operationalized by the interval between successive culturing days. Based on guidelines recommending obtaining blood cultures at intervals of 48 hours or 2 days until clearance [6, 9–11], daily culturing was considered more frequent than recommended.
2. **Obtaining >2 sets at the same day**, operationalized by the number of sets obtained per day. Two blood culture sets provide the recommended blood volume and diagnostic sensitivity for bloodstream infections [3, 4]. Additional sets have limited expected incremental diagnostic yield.
3. **Continuing culturing after 2 consecutive negative culturing days**, operationalized by the duration of follow-up culturing after the first negative set. Clearance was predefined as 2 consecutive negative culturing days (Table 1) to account for intermittent negative cultures (skip phenomenon) described in SAB [23, 24]. Continued culturing beyond this point was considered an opportunity for stewardship.

### Analysis

All analyses were performed in R version 4.5.2. Descriptive statistics summarized patient characteristics and blood culture timing relative to the index culture. The primary analysis assessed guideline adherence during the follow-up period. The number of follow-up blood culture sets, follow-up culturing, and duration of bacteremia days per episode were compared between MRSA versus methicillin-susceptible *S. aureus* (MSSA) and endocarditis versus non-endocarditis episodes using the Mann-Whitney U test, whereas proportions were compared using the chi-square test. For follow-up cultures, we quantified culturing timing, frequency, and opportunities for diagnostic stewardship using 3 operational metrics:

1. **Intervals between culturing days.** Within each episode, intervals were defined as the difference in calendar days between successive culturing days. The denominator consisted of the total number of measurable intervals across episodes. Intervals were calculated only when a subsequent culturing day was available; therefore, the final culturing day of an episode did not contribute an interval. Intervals were categorized as 1 day (daily), 2 days, 3 days, or ≥4 days. 1-day intervals were labeled as stewardship opportunity. Additionally, to explore the diagnostic value of closely spaced sets, we examined microbiological concordance between consecutive sets.
2. **Number of sets per culturing day.** On each culturing day, the first 2 sets were considered appropriate; additional sets were labeled as stewardship opportunity.
3. **Duration of follow-up culturing after the first negative set.** Within each episode, we identified the first negative follow-up day and counted consecutive negative days thereafter. A day was considered negative only if all bottles collected on that day showed no growth. Sets on the third or subsequent consecutive negative days were labeled as stewardship opportunity.

These 3 metrics were combined to classify sets meeting at least 1 criterion for a potential stewardship opportunity. We additionally assessed the occurrence of the skip phenomenon.

## RESULTS

### Study Population

During the 10-year study period, we identified 2400 adult patients with ≥1 *S. aureus*-positive blood culture. The median age of the cohort was 71 years, and 36.8% was female (Table 2). In total, 2499 SAB episodes were recorded for 2400 patients. Recurrence within an episode occurred in 25 patients (1.0%), and 82 patients (3.4%) had multiple episodes within the 10-year study period (median 2, range 2–8). Thirty-day mortality was 19.2% in Hospital A and 21.8% in Hospital C. The duration of bacteremia was slightly longer in MRSA than in MSSA episodes (median 2 [IQR 1–4] vs 1 [IQR 1–3] days, *P* = .047) and in patients with endocarditis compared to those without endocarditis (median 2 [IQR 1–4] vs 1 [1–3] days, *P* = .004).

**Table 2.** Dataset Characteristics

Characteristic	Hospital A	Hospital B	Hospital C	Total
Patients	594	548	1258	2400
Age**^a^**	64 [52–74]	75 [62–84]	72 [62–80]	71 [59–80]
Female sex**^a^**	217 (36.5%)	209 (38.1%)	456 (36.2%)	882 (36.8%)
SAB Episodes	634	566	1299	2499
Source of infection^b^				
Line-related	101 (19.3%)	NA	NA	NA
Skin and soft tissue	158 (30.2%)	NA	NA	NA
Osteoarticular	81 (15.5%)	NA	NA	NA
Endocarditis	35 (6.7%)	NA	NA	NA
Central nervous system	10 (1.9%)	NA	NA	NA
Other	245 (46.8%)	NA	NA	NA
Mortality^c^				
30-day	122 (19.2%)	NA	283 (21.8%)	NA
90-day	172 (27.1%)	NA	361 (27.8%)	NA
1 year	225 (35.5%)	NA	472 (36.3%)	NA
Duration of bacteremia^c^	1 [1–3] (1–18)	1 [1–3] (1–17)	1 [1–4] (1–12)	1 [1–3] (1–18)
MRSA	12 (1.9%)	11 (1.9%)	23 (1.8%)	46 (1.8%)
Blood culture bottles taken	9197	6967	25529	41693
Blood culture sets taken	4675	3546	12793	21014
Per episode	6 [4–9]	5 [4–8]	8 [4–14]	7 [4–11]

Continuous variables are presented as median [IQR]. For duration of bacteremia, the range is additionally reported in parentheses. Categorical variables are presented as n (%). SAB = *S. aureus* bacteremia. MRSA, methicillin-resistant *S. aureus*. NA, not available.

^a^Of first episode of *S. aureus* bacteremia.

^b^For Hospital A, infection foci were missing for 70 patients, only the absence of endocarditis had been recorded. These patients were excluded from the denominator. Multiple infection foci could be recorded per patient.

^c^Duration of bacteremia was defined as the number of calendar days from the first through the last positive *S. aureus* blood culture within a period of bacteremia. For this calculation, consecutive positive culture days separated by no more than 7 days were considered part of the same period of bacteremia.

### Blood Culture Sampling

The distribution of cultures performed after the index day is shown in Figure 1. No further blood cultures were obtained in 290 of 2499 episodes (11.6%) (Figure 1). Two potential explanations for the absence of further culturing days were evaluated: imminent death and transfer to another healthcare facility. Early death (<7 days after the index culture) accounted for 59.5% and 48.7% of these patients in Hospitals A and C, respectively; transfer explained an additional 21.4% in Hospital A (see Supplementary Table 1).

**Figure 1.**
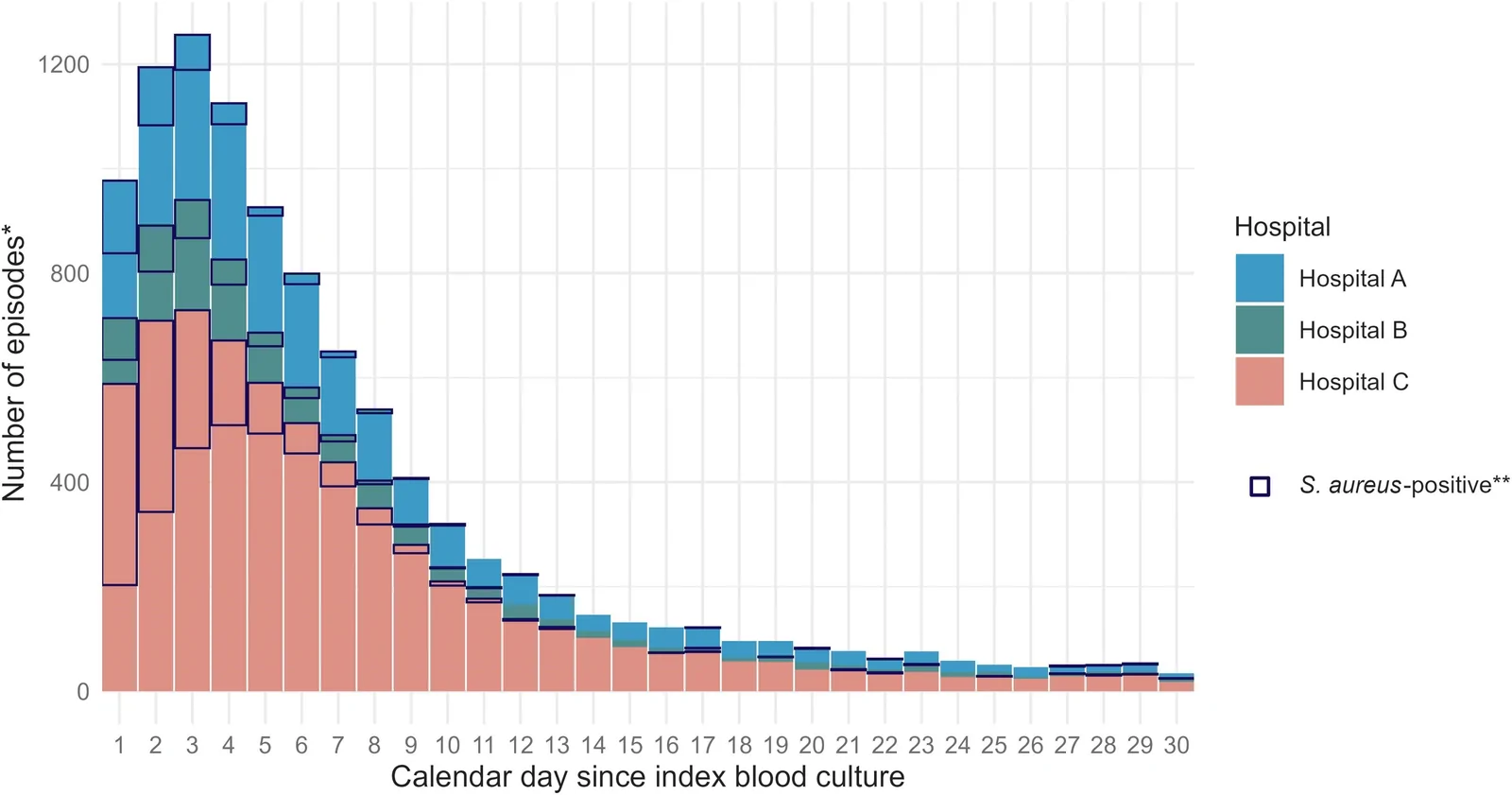
Distribution of calendar days per episode with ≥1 blood culture obtained after the index culture. The index blood culture day (day 0) is not shown; therefore, calendar days 1 and 2 fall within the diagnostic period, and the follow-up period starts on calendar day 3. *Episodes with ≥1 blood culture obtained on that calendar day. **Outlined segments indicate calendar days per episode that were classified as S. aureus-positive, defined as ≥1 blood culture bottle growing *S. aureus*.

Across all blood culture sets obtained during the SAB episodes, 1069 contained at least 1 non-*S. aureus* microorganism, either alone or in combination with *S. aureus* (Supplementary Figure 1).

### Time to Detection

Within the index culture set, 82.1% of *S. aureus*-positive sets had growth detected within 24 hours, 93.2% within 48 hours, 96.6% within 72 hours, 98.5% within 96 hours, and 99.7% within 120 hours. The median time to detection was 15.0 hours (IQR 11.6–20.7).

### Diagnostic Period

Overall, 8313 of 21014 blood culture sets (39.6%) were obtained during the diagnostic period. Across 2499 episodes, 253 (10.1%) had 1 set collected, 701 (28.1%) 2 sets, 451 (18.0%) 3 sets, and 1094 (43.8%) > 3 sets (median 3; IQR 2–4) during this period.

Among index blood culture sets, 319/4156 (7.7%) were polymicrobial. Coagulase-negative staphylococci (CoNS) (excluding *Staphylococcus lugdunensis)* or unspecified *Staphylococcus* spp. were identified in 81/319 (25.4%) of these polymicrobial index sets.

### Follow-up Period

Over the whole dataset, 12701 of 21014 blood culture sets (60.4%) were obtained during the follow-up period. After excluding episodes in which patients died before the start of the follow-up period (from hospitals with available mortality data: Hospitals A and C), follow-up blood cultures were performed in 1993 of 2389 episodes (83.4%). The median number of follow-up sets per episode was 4 (IQR 2–8). The median number of follow-up culturing days per episode was 3 (IQR 2–6). This was similar between MRSA and MSSA episodes (median follow-up sets: 4 [IQR 2–10] vs 4 [IQR 2–8], *P* = .58; median follow-up culturing days: 3 [IQR 2–7] vs 3 [IQR 2–6], *P* = .78). Notably, among Hospital A patients, patients with endocarditis had a more intensive follow-up than those without (median follow-up sets: 8 [IQR 4–12] vs 4 [IQR 2–6], *P* < .001; median follow-up culturing days: 6 [IQR 3–8] vs 3 [IQR 2–5], *P* < .001).

During follow-up, at least 1 non-*S. aureus* microorganism was identified in 515 of 12701 sets (4.1%) of which 209 (40.6%) were CoNS (excluding *S. lugdunensis*) or unspecified *Staphylococcus* spp.

#### (1) Intervals Between Culturing Days

Table 3 summarizes intervals between consecutive follow-up culturing days. A total of 6044 intervals were identified. Most were 1-day intervals (64.0%), indicating repeat culturing the next calendar day. Intervals of ≥4 days had a median duration of 7 days (IQR 5–12, range 4–27).

**Table 3.** Time Intervals Between Consecutive Follow-up Blood Culturing Days

Time Interval Category	Intervals
1 day (daily culturing)	3869/6044 (64.0%)
2 days	1022/6044 (16.9%)
3 days	328/6044 (5.4%)
≥4 days	825/6044 (13.6%)

Intervals were calculated between successive follow-up culturing days. Values are denoted as n/N (%); n represents the number of intervals and N the total number of intervals.

Overall, microbiological concordance between follow-up sets and the immediately preceding set predominated across all intervals. Concordance declined with increasing interval length and was highest (90.5%) when sets were obtained on the same day and remained high for sets obtained 1 day apart (85.3%) (Supplementary Table 2).

#### (2) Number of Sets per Culturing Day

A single or double set was obtained in 47.3% and 48.9% of the culturing days, respectively, while ≥3 sets were obtained in 3.8% (Supplementary Table 3). Among 3931 culturing days with 2 obtained sets, the second set provided additional information in 816 days (20.8%): in 754 (19.2%) due to discordant positivity and in 62 (1.6%) due to different microorganisms.

#### (3) Duration of Follow-up After the First Negative Set

Among 1993 episodes with follow-up blood cultures, 1902 (95.4%) had ≥1 negative follow-up culturing day. After the first negative day, the median number of consecutive negative culturing days was 1 (IQR 1–3, range 0–22). Follow-up culturing stopped immediately after the first negative set in 695/1993 episodes (34.9%). Another 395 (19.8%) had 1 additional negative day (ie, clearance). Longer sequences after clearance occurred in 903 episodes (45.3%) (Figure 2), contributing to 4231 blood culture sets. In episodes with extended culturing, 3 potential explanations were evaluated based on the available data: detection of non-*S. aureus* organisms, recurrent SAB, and management in critical care wards, where more extensive diagnostic work-up may occur. At least 1 identifiable explanation was present in 33.2% of prolonged sequences (see Supplementary Table 4).

**Figure 2.**
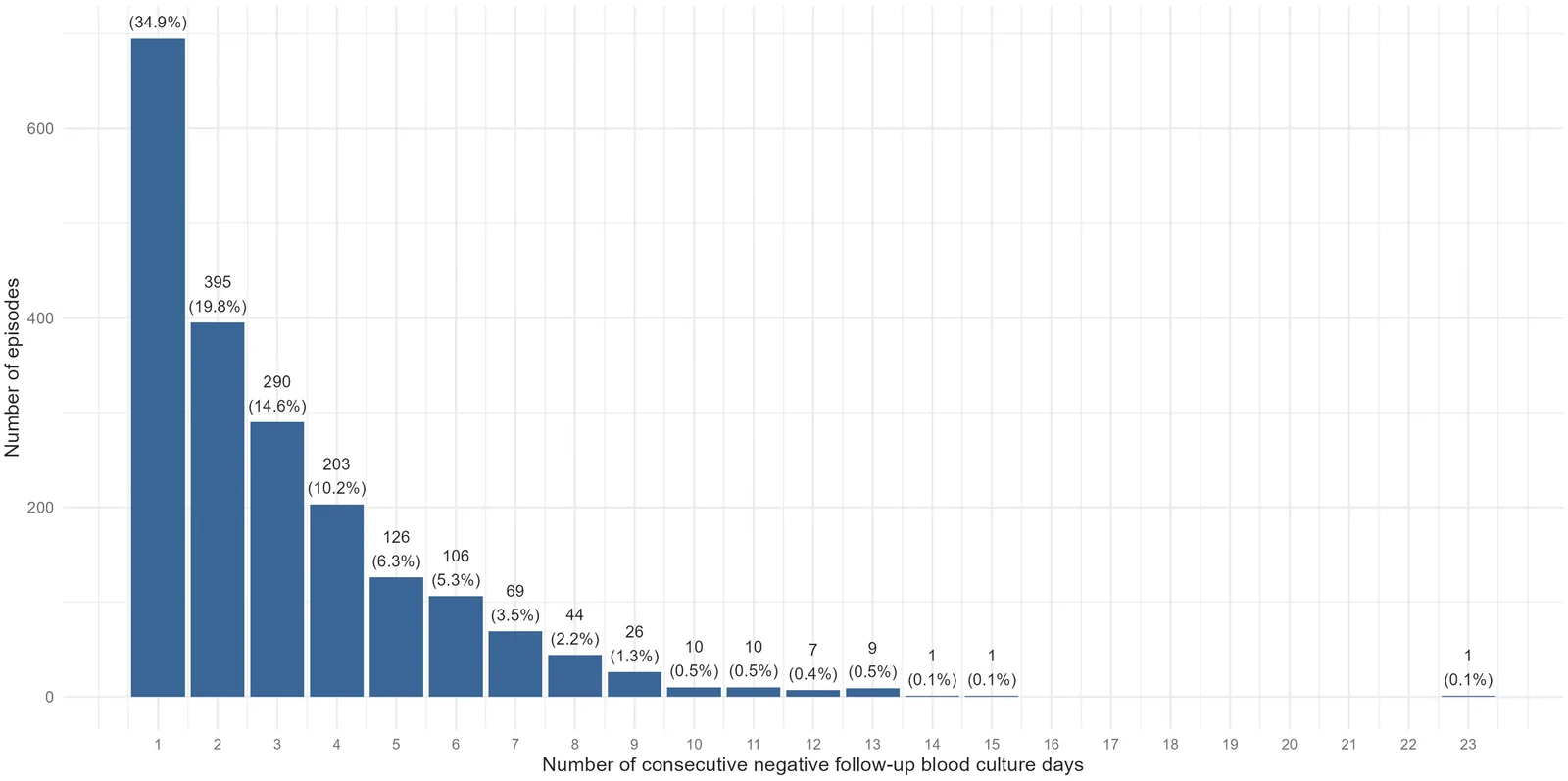
Consecutive negative follow-up culturing days. For each episode, the number of consecutive culturing days with exclusively negative follow-up blood cultures was counted, starting from the first negative culturing day. Counting stopped when a) a subsequent positive day occurred or b) when no further blood cultures were obtained. Percentages are calculated using a denominator of 1993 follow-up episodes.

#### Opportunities for Diagnostic Stewardship

Based on the 3 predefined metrics, 6432 of 12701 follow-up blood culture sets (50.6%) met at least 1 stewardship criterion (Figure 3). Among the 4033 follow-up blood culture sets obtained after 2 consecutive negative culturing days, 1282 (31.8%) originated from episodes with ≥1 of the potential explanations described above. This proportion differed between hospitals: 19.6% (Hospital B), 48.1% (Hospital A), and 58.7% (Hospital C) (Supplementary Table 5). Stratified by methicillin susceptibility, the proportion of culture sets meeting at least 1 stewardship criterion was similar in MRSA and MSSA episodes (55.2% vs 50.6%, *P* = .13). Among Hospital A patients, this proportion was significantly higher in patients with endocarditis than in those without endocarditis (61.3% vs 46.7%, *P* < .001). The higher proportion was mainly driven by more frequent culturing after clearance (40.1% vs 29.6%) and by daily culturing (30.8% vs 23.7%).

**Figure 3.**
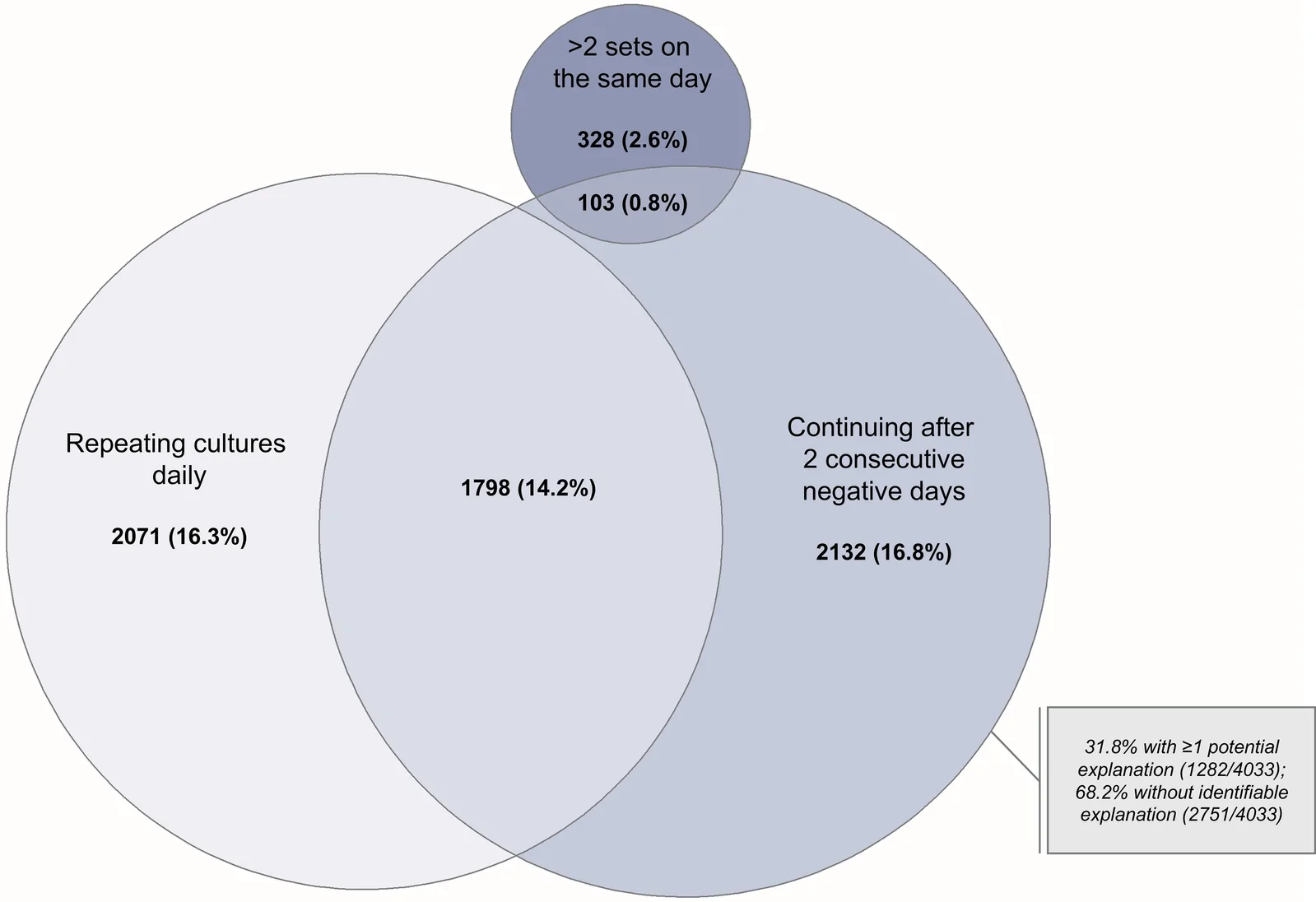
Diagnostic stewardship labels of follow-up blood culture sets. For this diagram, the denominator is the total number of follow-up blood culture sets (12701).

### Skip-phenomenon

To assess the skip phenomenon, the denominator consisted of episodes with sufficient data to evaluate this pattern. Episodes were considered evaluable if a *S. aureus-*positive day was followed by a negative culturing day and had at least 1 subsequent culturing day to assess renewed positivity. Among 1593 evaluable episodes, the skip phenomenon occurred in 120 (7.5%), accounting for 124 skip days. Of these skip days, 64 (51.6%) had a single set obtained, 57 (46.0%) a double set, and 3 (2.4%) ≥ 3 sets.

## DISCUSSION

This large multicenter study quantified follow-up blood culture practices in SAB patients and evaluated adherence to guideline recommendations and diagnostic stewardship opportunities. As advised in guidelines, follow-up blood cultures were performed in the majority (83.4%) of SAB patients known to be alive at the start of follow-up. However, 50.6% of all follow-up blood culture sets were potentially excessive as they met at least 1 predefined stewardship criterion. The largest contribution stemmed from continued culturing after 2 consecutive negative culturing days, closely followed by daily culturing, suggesting that stewardship should primarily target duration and frequency beyond guideline recommendations.

Several studies have addressed blood culture stewardship [22, 25, 26]. While the implementation of quality indicators has emphasized the need for follow-up cultures in SAB for documenting clearance, previous studies regarding guideline adherence mainly addressed whether any follow-up cultures were obtained at all [27–30]. Only 1 study evaluated timing and reported correct 72-hour follow-up in 65% of the cases, without assessing additional cultures or documentation of negativity [31]. Our findings extend this literature by demonstrating that once follow-up cultures are initiated, they are often obtained more frequently and for longer than recommended. A recently published Dutch study similarly identified substantial potential for reducing follow-up blood culturing in SAB, reporting that after a negative follow-up culture within 48 hours, persistent bacteremia would have been potentially missed in 1.0% of patients without clinical triggers for further culturing [32]. Whereas their study focused on early negative follow-up cultures and the skip phenomenon, our study evaluated the frequency and duration of follow-up culturing throughout the SAB episode, thereby broadening the evidence base for diagnostic stewardship.

Our findings also provide microbiological support for the safety of reducing follow-up culturing with respect to underdetection of persistent *S. aureus* bacteremia, complementing evidence that a clinically guided approach after early negative follow-up cultures carries a low risk of missing persistent bacteremia [32]. Two findings from our study are particularly relevant: (1) the diagnostic value of closely spaced follow-up sets, and (2) prolonged culturing after clearance.

First, we assessed the diagnostic value of closely spaced follow-up sets by examining microbiological concordance between consecutive sets. Concordance was high for sets obtained on the same day (90.5%) and remained high for sets obtained 1 day apart (85.3%). These findings suggest limited additional microbiological information from very frequent follow-up culturing.

Second, we evaluated prolonged follow-up culturing after clearance, which occurred in 45.3% of episodes. Interpretation of prolonged follow-up culturing after clearance requires consideration of the 5-day incubation period [12–14]. Because cultures are not considered definitively negative until sufficient incubation time has elapsed, some repeat cultures may have been obtained while earlier sets were still incubating. Furthermore, prolonged culturing may have been justified by new signs of infection or the detection of non-*S. aureus* microorganisms.

In our cohort, we could not study new clinical signs of infection. However, additional microorganisms were found in 24.1% episodes with prolonged culturing after *S. aureus* clearance. In these cases, there may be an overestimation of stewardship labeling; nevertheless, these cases would not be the intended target of standardized stewardship interventions. Another reason for prolonged culturing could be a severe infection such as endocarditis or MRSA bacteremia, likely reflecting the greater concern of persistent bacteremia. Previous literature, however, showed that follow-up culturing after endocarditis has low yield [33]. Although bacteremia persisted significantly longer in patients with MRSA and endocarditis in our cohort, consistent with previous literature [34], this does not contradict our stewardship findings: the predefined stewardship criteria only identified potentially excessive culturing after clearance has been achieved. Taken together, our findings support obtaining 2 sets of follow-up blood cultures every 2 days until clearance, while avoiding further routine culturing thereafter in the absence of new clinical indications.

Reducing unnecessary follow-up blood cultures may decrease repeated phlebotomy and healthcare costs. In our cohort, based on all blood culture sets meeting at least 1 stewardship criterion, this would correspond to an average reduction of approximately 65 mL of blood per episode (assuming 20 mL of blood per set) and approximately €13 000 per 100 SAB episodes (based on the Dutch 2026 publicly available tariff of approximately €40 per blood culture [35]). Several diagnostic stewardship interventions, including education, audit and feedback, and electronic record algorithms, have been shown to safely and effectively reduce blood culture use [21, 25]. Clinician education regarding appropriate follow-up culturing may be a practical strategy to improve adherence to guideline recommendations while ensuring that clinically indicated blood cultures are still obtained.

While our findings identify opportunities to reduce unnecessary follow-up culturing, they also highlight the importance of obtaining sufficient cultures to reliably document clearance. Follow-up culturing stopped after the first negative set in 34.9% of episodes, indicating that in a substantial proportion of episodes clearance was not confirmed. In our cohort, the skip phenomenon occurred in 7.5% of the episodes in which this pattern could be assessed. This is in line with earlier studies that reported skip rates between 4.0% and 13.3% [7, 23, 24, 36]. Notably, 51.6% of the skip days in our study involved a single set. At the same time, nearly half of all follow-up culturing days consisted of a single set (47.3%). On days where a double set was obtained, discordant results between sets were observed in 20.8% of cases. Together, these findings support obtaining 2 sets per culturing day to reliably detect persistent or intermittent bacteremia, consistent with previous literature recommendations [4, 7, 25, 37]. These findings therefore support a stewardship strategy of less frequent but still complete culturing: when follow-up cultures are indicated, 2 sets should be obtained.

Although this study primarily focused on follow-up blood cultures, we also observed that 43.8% of episodes had >3 sets obtained during the diagnostic period, exceeding commonly recommended benchmarks for suspected bacteremia or endocarditis [3–5, 38]. Importantly, as the diagnostic period was defined pragmatically as the first 2 days, some cultures classified as diagnostic may represent early follow-up cultures. Regardless if they are diagnostic or follow-up cultures, these blood cultures could be additional stewardship targets.

Strengths of this study include the large multicenter cohort, which enhances generalizability, and a long observation period of 10 years. Uniform definitions ensured clear and consistent classification of blood cultures. However, several limitations should be acknowledged. First and most importantly, our data cannot determine the clinical appropriateness of individual decisions. The optimal frequency and duration are likely influenced by clinical factors including endocarditis, other deep-seated foci, source control, MRSA, immunosuppression, and the timing of effective antimicrobial therapy. With the exception of focus of infection (for Hospital A) and MRSA, these data were unavailable in the present multicenter dataset and therefore could not be incorporated into our analyses. Therefore, prolonged culturing may also reflect suspected persistent or recurrent SAB (although recurrences were low in our study) or suspected other bacterial infections (pathogens other than CoNS were identified in 13.5% of episodes with prolonged culturing after clearance). We attempted to account for this by identifying episodes with potential explanations for extended culturing, but this could either be an over- or underestimation. Our predefined stewardship criteria were intended to identify potential opportunities for diagnostic stewardship at an aggregate level, but we cannot establish that individual episodes represented (in)appropriate culturing. In addition, as this is a descriptive study based on cultures obtained as part of routine clinical practice, we could not directly compare different follow-up culturing strategies or prospectively assess safety; our assessment was therefore limited to microbiological measures.

Second, the time at which clinicians became aware of culture results was unknown; time to detection was only available in Hospital A and does not reflect communication time, which may influence culturing decisions. Third, culture (clock) time could not be reliably used due to inconsistent recording, requiring the use of calendar days as the unit of analysis, increasing the risk of potential misclassification. Fourth, the long observation period may introduce temporal variation, as local protocols may have changed over time. In fact, in hospital C the local protocol prescribed daily follow-up culturing, although this is no longer current practice. Our analyses therefore reflect average practices over the 10-year period.

To conclude, in our study, follow-up culturing for SAB was often not aligned with guideline recommendations. Our findings highlight an opportunity for diagnostic stewardship by obtaining 2 sets of follow-up blood cultures every 2 days until clearance.

## Supplementary Material

ofag685_Supplementary_Data

## Supplementary Data

Supplementary materials are available at *Open Forum Infectious Diseases* online. Consisting of data provided by the authors to benefit the reader, the posted materials are not copyedited and are the sole responsibility of the authors, so questions or comments should be addressed to the corresponding author.
