# Supplementary material for: Potentially Excessive Follow-up Blood Cultures in Staphylococcus aureus Bacteremia: Opportunities for Diagnostic Stewardship
Source: Open Forum Infect Dis. 2026 Oct 29;13(11):ofag685. doi: 10.1093/ofid/ofag685 (PMC13756073; doi:10.1093/ofid/ofag685)
Supplement: Supplementary file 1 [file ofag685_supplementary_data.docx]

Supplementary materials

Index

[Supplementary Table S1. Possible reasons for absence of follow-up blood culturing after the index culturing day.	2](#_Toc235106437)

[Supplementary Figure S1. Non-*S. aureus* microorganisms, for sets where *S. aureus* was or was not identified.	3](#_Toc235106438)

[Supplementary Table S2. Microbiological concordance of follow-up blood culture sets relative to preceding set.	4](#_Toc235106439)

[Supplementary Table S3. Number of blood culture sets per follow-up day.	5](#_Toc235106440)

[Supplementary Table S4. Possible explanations for episodes with >2 consecutive negative days.	6](#_Toc235106441)

[Supplementary Table S5. Stewardship proportions stratified by hospital.	7](#_Toc235106442)

# Supplementary Table S1. Possible reasons for absence of follow-up blood culturing after the index culturing day.

| Hospital | Total patients | Patients without further culturing | Early mortality (within 7 days) | Transfer | Explained by mortality/transfer |
| --- | --- | --- | --- | --- | --- |
| Hospital A | 594 | 42/594 (7.2%) | 25/42 (59.5%) | 9/42 (21.4%) | *Mortality + transfer*  34/42 (81.0%) |
| Hospital B | 548 | 84/548 (15.3%) | NA | NA | *NA* |
| Hospital C | 1258 | 152/1258 (12.1%) | 74/152 (48.7%) | NA | *Mortality only*  74/152 (48.7%) |
| Total | 2400 | 278/2400 (11.6%) | NA | NA | NA |

NA (not available) because data was unavailable for that hospital.

# Supplementary Figure S1. Non-*S. aureus* microorganisms, for sets where *S. aureus* was or was not identified.

# Supplementary Table S2. Microbiological concordance of follow-up blood culture sets relative to preceding set.

| Time to next set | Sets | Same result as preceding set | Both sets *S. aureus* positive |
| --- | --- | --- | --- |
| 0 days | 4664 | 4222 (90.5%) | 489 (10.5%) |
| 1 day | 4559 | 3888 (85.3%) | 515 (11.4%) |
| 2 days | 1486 | 1125 (75.7%) | 168 (11.3%) |
| 3 days | 790 | 434 (54.9%) | 101 (12.8%) |
| ≥4 days | 1202 | 824 (68.6%) | 31 (2.6%) |
| *Total* | *12701* | *10493 (82.6%)* | *1304 (10.3%)* |

Values are denoted as n (%). Same result indicates identical microbiological outcome between the sets (e.g., negative-negative or positive-positive for the same microorganism).

# Supplementary Table S3. Number of blood culture sets per follow-up day.

| Category of sets | Follow-up culturing days |
| --- | --- |
| 1 set | 3804/8037 (47.3%) |
| 2 sets | 3931/8037 (48.9%) |
| ≥3 sets | 302/8037 (3.8%) |

Values are denoted as n/N (%); n represents the number of follow-up culturing days in each category, and N the total number of follow-up culturing days.

# Supplementary Table S4. Possible explanations for episodes with >2 consecutive negative days.

| Hospital | Total episodes | Episodes with >2 consecutive negative days | Non-*S. aureus* organism detected^a^ | Recurrent SAB^a^ | Critical care admission^a^ | Episodes with ≥1 identifiable explanation^b^ |
| --- | --- | --- | --- | --- | --- | --- |
| Hospital A | 634 | 270/634 (42.6%) | 61/270 (22.6%) | 5/270 (1.9%) | 94/270 (34.8%) | 121/270 (44.8%) |
| Hospital B | 566 | 65/566 (11.5%) | 4/65 (6.2%) | 1/65 (1.5%) | NA | 5/65 (7.7%) |
| Hospital C | 1299 | 568/1299 (43.7%) | 153/568 (26.9%) | 10/568 (1.8%) | 30/568 (5.3%) | 174/568 (30.6%) |
| Total | 2499 | 903/2499 (36.1%) | 218/903 (24.1%)^c^ | 16/903 (1.8%) | 124/903 (13.7%) | 300/903 (33.2%) |

**^a^**Categories are not mutually exclusive. ^b^Unique episodes with at least one identifiable explanation. ^c^Of the episodes with a non-*S. aureus* organism, coagulase-negative staphylococci (CNS) (excluding *S. lugdunensis*) or unspecified *Staphylococcus* spp. accounted for 96/218 (44.0%). Therefore, of the total episodes with prolonged culturing after clearance, detection of a non-CNS microorganism as possible explanation accounted for 122/903 (13.5%) episodes.

# Supplementary Table S5. Stewardship proportions stratified by hospital.

| Hospital | Daily culturing (%) | >2 sets/day (%) | Continuing after 2 consecutive negative days (%) | Total (≥1 stewardship criterion) (%) |
| --- | --- | --- | --- | --- |
| Hospital A | 24.4 | 4.5 | 30.6 | 48.1 |
| Hospital B | 7.2 | 2.7 | 11.6 | 19.6 |
| Hospital C | 38.0 | 3.2 | 36.8 | 58.7 |
